# An approach to hypertensive disorders in pregnancy for the primary care physician

**DOI:** 10.4102/safp.v62i1.5095

**Published:** 2020-02-17

**Authors:** Mergan Naidoo, Robert C. Pattinson

**Affiliations:** 1 Department of Family Medicine, College of Health Sciences, University of KwaZulu-Natal, Durban, South Africa; 2 Department of Obstetrics and Gynaecology, Faculty of Health Sciences, University of Pretoria, Pretoria, South Africa

**Keywords:** hypertensive disorders in pregnancy, primary care, management

## Abstract

Hypertensive disorders in pregnancy (HDP) are a leading obstetric cause for maternal morbidity and mortality nationally as well as globally. The Saving Mothers is a report published every three years by the National Committee for Confidential Enquiry, which reports the trends in maternal deaths in South Africa. The last three Saving Mothers reports identified many gaps in the management of HDP and interventions to address these gaps were recommended. The recently published national guidelines on the management of HDP have highlighted approaches for the diagnosis, assessment and management of HDP. This article synthesises the national guidelines and provides approaches for the primary care physician working at the primary healthcare or the district hospital level. The algorithms provide easy clinical pathways once the correct assessment has been made.

## Introduction

The recently published guidelines on the management of hypertensive disorders in pregnancy (HDP) highlighted the concerning trends in maternal deaths in South Africa. Although 78% of deaths occurred at higher levels of care, many of the emergencies are thought to have originated at the primary healthcare (PHC) or the district hospital (DH) level.^1^ The guidelines further highlight the large percentage (75%) of potentially preventable deaths because of HDP at all levels of care, which has been increasing over the last decade.^1^ Avoidable factors for HDP deaths were identified in 48% and 60% of the cases at the Community Health Centre (CHC) and DH level, respectively.^1^ The factors identified at a PHC and DH included inadequate assessment, errors in diagnosis, delayed or no referrals to higher levels of care, non-adherence to management protocols, poor monitoring and poor response to abnormal monitoring.^1^ These guidelines provide an approach to diagnosing, assessing and managing HDP at PHC and DH levels and are based on the national guidelines published in 2019.

## Diagnosis of hypertensive disorders in pregnancy

Diagnosis of HDP is based on the classification of the International Society for the Study of Hypertension in Pregnancy (ISSHP)^2^:

1. Chronic hypertension (HT) may predate the pregnancy or is diagnosed before 20 weeks’ gestation.
2. White-coat HT refers to elevated clinical (≥ 140/90 mmHg) blood pressure (BP), but normal BP measured at home may convey increased risk for pre-eclampsia (PE).
3. Masked HT is characterised by BP that is normal at the clinical level but elevated at other times, and it is diagnosed by 24-h ambulatory BP monitoring or automated home BP monitoring.
4. Gestational HT arises after 20 weeks’ gestation in the absence of proteinuria and is not usually associated with foetal growth restriction or poor pregnancy outcomes.
5. Pre-eclampsia is diagnosed by the presence of HT after 20 weeks’ gestation accompanied by proteinuria or evidence of maternal acute kidney injury, liver dysfunction, neurological features, haemolysis or thrombocytopenia, or foetal growth restriction. Pre-eclampsia may develop or can be recognised for the first time intrapartum or early postpartum. The haemolysis, elevated liver enzymes, and low platelets (HELLP) syndrome is a (serious) manifestation of PE and is not a separate disorder.

### Measurement of blood pressure

Blood pressure should be measured with the woman in a relaxed sitting position keeping the arm at the level of the heart with an appropriately sized cuff (1.5 times the circumference of the arm). Korotkoff Phase 5 (K-5) should be used to designate diastolic BP. If the BP is consistently higher in one arm, then the arm with higher values should be used for all BP measurements. Blood pressure should be measured using a validated device. Hypertension in pregnancy is defined as a clinical systolic BP ≥ 140 mmHg or diastolic BP ≥ 90 mmHg, based on the average of at least two measurements taken at least 15 minute apart, using the same arm.^1,2,3^ Patients presenting with pre-HT (BP = 135/85–139/89) should have their BP repeated within 2 h and if BP is still at borderline, they should be asked to return within 3–7 days.^1^

### Assessment of patients with hypertensive disorders in pregnancy

A detailed history and examination of the patient is warranted at first presentation. This would include a history of prior PE, chronic HT, diabetes mellitus, anti-phospholipid syndrome, systemic lupus erythematous, previous pregnancy outcomes and the use of assisted reproduction therapies. Examination may reveal multiple gestation and a high maternal body mass index (BMI > 35). Patients with any of these findings during history and examination should be started on prophylactic aspirin (75 mg - 162 mg) in early pregnancy with maximum benefits in preventing PE realised when initiated < 16 weeks of gestation.^1,2,4,5^ All pregnant women should also be started on elemental calcium, minimum 500 mg daily, as prophylaxis for PE.^1,2,5,6,7,8^

Table 1 provides investigations that are to be carried out at various antenatal visits.^1,2,3^

**TABLE 1:** Recommended investigations during antenatal care.

Investigation	When	Why
Urine dipsticks	At every visit	To confirm the presence of proteinuria and make a diagnosis of PE
Serum creatinine	When a diagnosis of essential or gestational HT or PE with no severe features is made	To establish renal damage
Serum haemoglobin and platelets	When a diagnosis of essential or gestational HT or PE with no severe features is made	To confirm intravascular depletion
Ultrasound examination	When a diagnosis of essential or gestational HT or PE with no severe features is made	To establish foetal well-being
PCR or 24-hour urinary protein excretion	When PE with no severe features is diagnosed	To estimate the amount of protein excreted in urine
Urine microscopy, culture and sensitivity	When PE with no severe features is diagnosed	To exclude an alternative cause of proteinuria
ALT	When PE with no severe features is diagnosed	To confirm liver involvement
Urea and electrolytes, liver function tests, INR, serum uric acid levels, full blood count, crude clotting time	When PE with severe features is diagnosed	To evaluate organ system involvement. Do not delay transfer waiting for investigations
Arterial blood gas	When pulmonary oedema is suspected	To ascertain need for assisted ventilation
Uterine artery Doppler velocimetry	When placental insufficiency is suspected in a patient with HDP	To exclude foetal compromise

*Source:* Please see the full reference list of the article, Moodley J, Soma-Pillay P, Buchmann E, Pattinson R. Hypertensive disorders in pregnancy: 2019 National guideline. S Afr Med J. 2019;109(9):12723, for more information

ALT, alanine aminotransferase; HDP, hypertensive disorders in pregnancy; HT, hypertension; INR, international normalised ratio; PE, pre-eclampsia; PCR, protein-creatinine ratio.

### Defining the next level of expertise and referral patterns

It is important for primary care providers to define the next level of expertise and the referral patterns. Primary healthcare clinics, CHCs and staff in some DHs need to identify the next level of expertise, which may include facilities that have an advanced midwife with special training or a doctor with expertise in managing pregnant women, and this facility should be able to perform basic laboratory investigations (defined in Table 1) and offer a basic obstetric sonar examination. The blood investigations must be processed rapidly so that management could be planned and an efficient communication network with obstetricians must be in place. These high-risk clinics may be present at CHCs and DHs. The referral routes for an identified catchment area should be defined by specialists based at the regional or tertiary hospitals in consultation with the district clinical specialist team (DCST). The main referral hospital should develop and circulate guidelines and protocols to all facilities in the catchment area. This helps to speed up referral through pathways and allows for the management of patient according to defined protocols. Discussions between staff at the PHC clinic, DH, DCST and the regional hospital (RH) help to define the level of expertise required at each level of care and the type of patient that could be managed at these levels. The emergency medical response service must be involved in these discussions as they are key in facilitating transfer across facilities.

### Management of patients with hypertensive disorders in pregnancy

**FIGURE 1:**
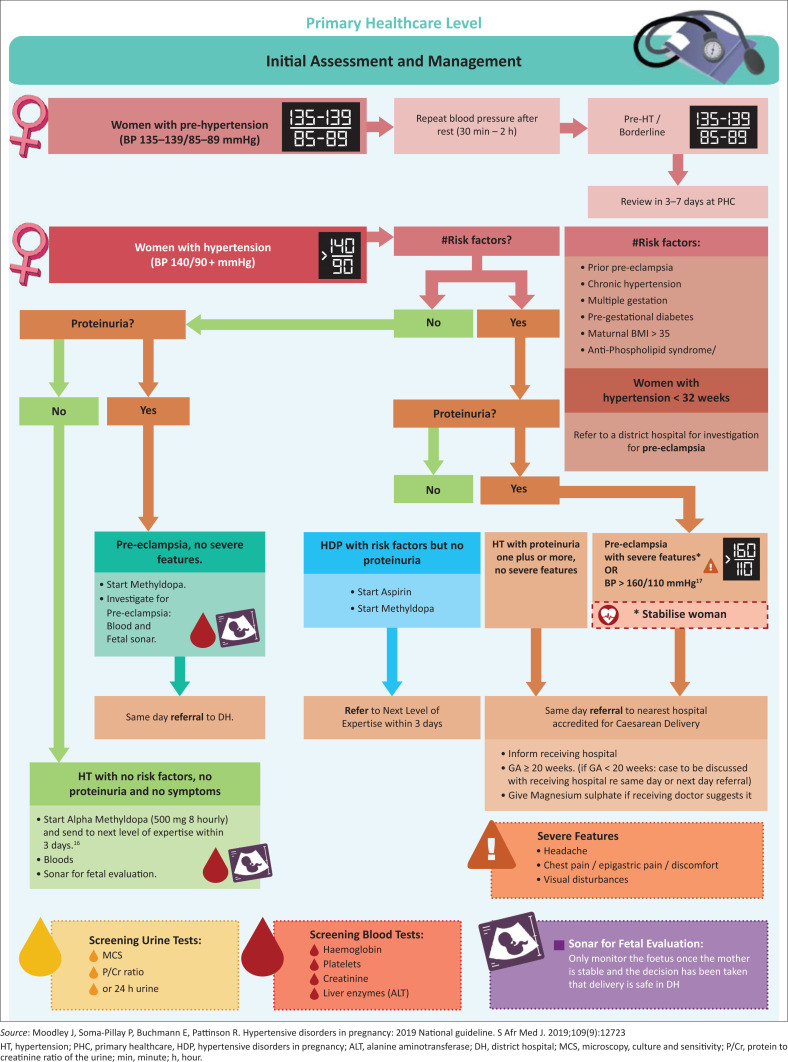
Management of a patient with hypertensive disorders in pregnancy at a primary healthcare clinic.

**FIGURE 2:**
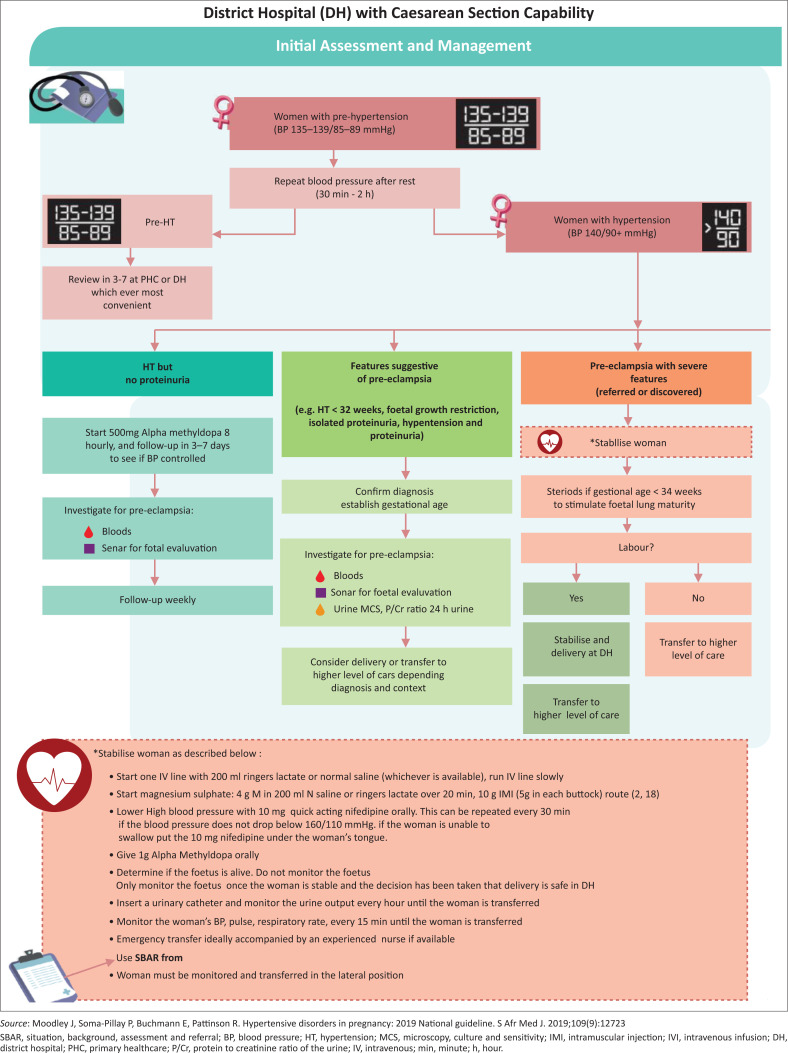
Management of a patient with hypertensive disorders in pregnancy at a district hospital. Standardised referral form used by public healthcare facilities in the Republic of South Africa.

**FIGURE 3:**
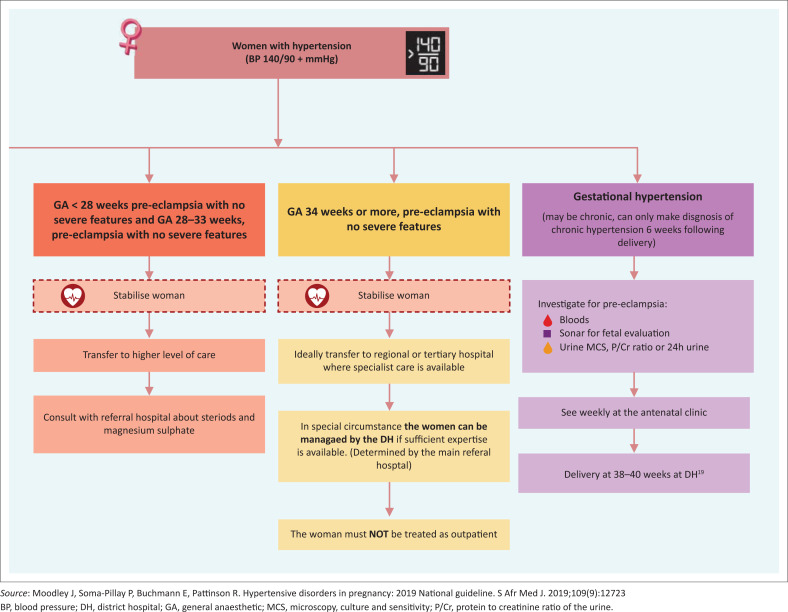
Management of patients with hypertensive disorders in pregnancy according to gestational age.

Figures 1–3 have been extrapolated from the South African guidelines on managing HDP and have been included as easy reference aids.^1^

Patients with PE and 1+ proteinuria with no severe features and BP that is controlled with one oral agent may be managed at a DH with caesarean section facilities, but these patients require *inpatient management*. It is important to inform the obstetrician at the RH that such a patient is being managed at the DH.^1^

## Management of pre-eclampsia with severe features

These patients may present to any facility and require emergency management. Such patients may present with headache, chest or epigastric pain or discomfort, visual disturbances, proteinuria 2+ or more and BP more than 160/110 mmHg. If the patient is at a PHC clinic or a CHC, one member of the team should inform the regional or tertiary referral hospital whilst other members should stabilise the patient according to the principles of resuscitation based on the Essential Steps in Managing Obstetric Emergencies (ESMOE), which follows a structured approach.^9^ An intravenous line of ringer’s lactate running in at 80 mL/h should be started, and the patient should be loaded with magnesium sulphate 14 g (4 g intravenous infusion [IVI] in 200 mL of normal saline over 20 min and 10 g given as intramuscular injection [IMI] – 5 g in each buttock).^1^ The BP could be brought down with 10 mg of oral nifedipine and a start dose of 1000 mg of oral alpha methyldopa. Emergency transfer to RH with monitoring by an experienced nurse during transit should take place with the patient continuously nursed in the lateral position.

Patients presenting with PE with severe features at a DH with caesarean delivery (CD) facilities are managed in a similar manner, but some DHs have access to intravenous labetalol and this could be used according to the standard protocol.^10^ Many women die because of injudicious use of excessive fluids, so careful monitoring of fluid intake is important, with the recommended rate of administration of IVI fluids not exceeding 80 mL/h. Women with a gestational age of 28–34 weeks should be given steroids to improve the lung maturity of the foetus, especially if delivery is planned within 48 h. The first dose of steroids should be given at the DH, and the patient should be urgently transferred to a RH.

### Management of pre-eclampsia with severe maternal complications

Severe maternal complications in PE refer to a patient who presents with eclampsia, pulmonary oedema, cerebrovascular accident, HELLP syndrome, renal dysfunction (serum creatinine > 120 mmol/L), severe uncontrolled HT and coagulopathy. Such patient needs urgent delivery after stabilisation. There are still significant shortcomings in the management of these life-threatening emergencies because of lack of skills and knowledge as evidenced in two local studies.^11,12^ The management of eclampsia should be based on the National Maternity Care Guidelines, a summary of which is presented below.^13^

Call for help and turn the patient on her side, extend her neck, suction the airway and insert an oral airway if possible. Administer oxygen through facemask and initiate magnesium sulphate according to the regimen listed above to arrest and prevent seizures. Start maintenance dose of magnesium sulphate, 4.5 g given by deep IMI every 4 h in alternative buttocks. If systolic or diastolic BP is > 160/110 mmHg, then use nifedipine orally, or labetalol intravenously. Nifedipine can be repeated after 30 min if BP remains > 160/110 mmHg. All patients should also be given 1000 mg methyldopa, with a repeated dose of 500 mg every 8 h orally. This is to ensure smoother control of BP at a later stage. Methyldopa is not a rapidly acting antihypertensive; hence, nifedipine or labetalol must be used in acute phase to control BP. If the patient is restless, administer 1 mg clonazepam as slow IVI.

After stabilisation, obtain full history and follow the ESMOE steps of evaluating the Big 5 (central nervous, respiratory, cardiovascular, liver and gastrointestinal and renal systems), Forgotten 4 (haematological, endocrine, immunological and musculo-skeletal systems) and Core 1 (female genital system).^9^ Monitor BP every 15 min. Assess the uterus for tenderness, irritability, foetal size and liquor volume. Moreover, assess the cervix to check whether induction of labour is needed. Draw blood for haemoglobin, platelet count, creatinine, alanine aminotransferase and lactate dehydrogenase (LDH). Assess the foetal condition if the patient is completely stable and the platelet count is known. Ensure that abruptio placenta is ruled out. Confirm whether the patient is in labour, as eclamptic patients often go into spontaneous labour. If vaginal delivery is not contraindicated, go for spontaneous vaginal delivery before transfer, but ensure that there is no excessive bearing down. Only use oxytocin, 10 units IMI, for active delivery of the placenta and for prevention of postpartum haemorrhage. Advice must be obtained from an experienced obstetrician and ensure that detailed notes are kept. If the patient is not in labour and is stable, transfer her to a specialist level of care. Continue monitoring the patient whilst awaiting transfer, preferably every 15 min. Anaesthesia for patients who need urgent CD is complex and should preferably be given by an experienced anaesthetist.^13^

## Conclusion

Managing HDP requires knowledge and skill, and the best way to obtain such competencies is through educational initiatives such as ESMOE. Participating in regular emergency obstetric simulations (fire drills) at the workplace allows primary care providers the opportunity to identify gaps in their knowledge and skills, and recognise and address shortcomings in equipment and drug stocks. The South African Academy of Family Physicians conducts interactive web-based educational programmes, and healthcare providers are strongly encouraged to participate in these activities.
